# Extracellular Vesicles in Chronic Obstructive Pulmonary Disease

**DOI:** 10.3390/ijms17111801

**Published:** 2016-10-27

**Authors:** Tsukasa Kadota, Yu Fujita, Yusuke Yoshioka, Jun Araya, Kazuyoshi Kuwano, Takahiro Ochiya

**Affiliations:** 1 Division of Molecular and Cellular Medicine, National Cancer Center Research Institute, Tokyo 104-0045, Japan; tskadota@ncc.go.jp (T.K.); yuugot@gmail.com (Y.F.); yyoshiok@ncc.go.jp (Y.Y.); 2 Division of Respiratory Disease, Department of Internal Medicine, The Jikei University School of Medicine, Tokyo 105-8461, Japan; araya@jikei.ac.jp (J.A.); kkuwano@jikei.ac.jp (K.K.)

**Keywords:** COPD, extracellular vesicles, exosome, microRNA, microvesicle, pathogenesis, biomarker, therapy, exacerbation, endothelial microparticle

## Abstract

Chronic obstructive pulmonary disease (COPD) is characterized by the progression of irreversible airflow limitation and is a leading cause of morbidity and mortality worldwide. Although several crucial mechanisms of COPD pathogenesis have been studied, the precise mechanism remains unknown. Extracellular vesicles (EVs), including exosomes, microvesicles, and apoptotic bodies, are released from almost all cell types and are recognized as novel cell–cell communication tools. They have been shown to carry and transfer a wide variety of molecules, such as microRNAs, messenger RNAs, and proteins, which are involved in physiological functions and the pathology of various diseases. Recently, EVs have attracted considerable attention in pulmonary research. In this review, we summarize the recent findings of EV-mediated COPD pathogenesis. We also discuss the potential clinical usefulness of EVs as biomarkers and therapeutic agents for the treatment of COPD.

## 1. Introduction

Chronic obstructive pulmonary disease (COPD) is an inflammatory and age-related lung disease characterized by a poorly reversible airflow limitation and is caused by inhaled cigarette smoke and other noxious particles [1,2]. COPD is a major condition that imposes a high disease burden and mortality worldwide. It is now estimated that more than 300 million people worldwide are affected by COPD, and of the 68 million deaths worldwide in 2020, 4.7 million people will die from the disease [1,3,4,5]. The pathologic hallmarks of COPD are characterized by the emphysematous destruction of the alveolar structure and the remodeling and narrowing of small airways [1,6]. Unfortunately, although several crucial mechanisms of COPD pathogenesis have been studied, the precise mechanism is incompletely understood. In addition, recent advances in the treatment of COPD, such as long-acting muscarinic antagonists and long-acting β2-adrenergic agonists, have demonstrated a certain degree of clinical efficacy [1]. However, a complete cure is unachievable with these currently available therapies. In light of this, there is a critical need to improve the understanding of COPD pathogenesis and identify a new therapeutic target.

Extracellular vesicles (EVs) include a wide variety of membrane-bound vesicles, ranging from approximately 30 nm to a few micrometers in size, which are released into the extracellular environment by almost all cell types. The presence of membrane-bound vesicles outside cells was recognized over 40 years ago [7,8]. At that time, direct shedding from the plasma membrane was assumed to be the only mechanism consider for secretion of these vesicles. However, in 1983, the groups of Philip Stahl and Rose Johnstone discovered that small membrane vesicles are also released by multivesicular bodies (MVBs) fusing with the cell membrane by using pulse-chase and electron microscopy experiments [9]. In 1987, Johnstone proposed to define such vesicles as exosomes [10]. At present, EVs can be categorized as exosomes, microvesicles (also known as microparticles), and apoptotic bodies according to their size, biogenesis, and secretion mechanisms [11,12,13]. Exosomes are defined as approximately 100 nm-sized vesicles surrounded by a phospholipid membrane. They are generated by the inward and reverse budding of an endosomal membrane and become MVBs that contain intraluminal vesicles (ILVs). Exosomes are released into the extracellular space by the fusion of the peripheral membrane of the MVBs with the limiting plasma membrane. Their cargo has proteins from the plasma membrane, the endosomes, the cytosol, and specific subsets of cellular proteins depending on the parent cell type [14,15,16]. Microvesicles, which are larger in size than exosomes, are generated from the plasma membrane by shedding or budding in normal circumstances or upon stimuli. Microvesicles are rich in phosphatidylserine and contain membrane components similar to those of the parent cell membrane [13]. Apoptotic bodies are a few µm in diameter and are released from the plasma membrane during cell apoptosis via indiscriminate blebbing of the plasma membrane [11,12,13,17]. Apoptotic bodies contain proteins from the plasma membrane and the cytosol, as well as fragmented nuclei [18]. Although the origins of exosomes, microvesicles, and apoptotic bodies have been defined, current technologies cannot clearly distinguish the different types of EVs. Thus, in this review, we use the term EVs according to the recommendations of the International Society for Extracellular Vesicles (ISEV) as a general term for all types of vesicles in the extracellular space [19]. In some sections, we supplementarily mention the vesicle types being discussed when the referenced studies specified them.

Recently, EVs have emerged as novel mediators of intercellular communication through the transfer of their contents. EV contents, which include proteins, messenger RNA (mRNA), microRNA (miRNA), DNA, lipids and metabolites [13,20], can be delivered to various sites in the body and influence a wide variety of biological processes of the recipient cells [21]. Interestingly, EVs are susceptible to and modified by environmental stressors. Indeed, stress conditions such as heat shock, hypoxia, hypothermia, oxidative stress, irradiation, and changes in conditioned media induce remarkable changes in the compositions of EVs, which can, in turn, modulate the stress response [22,23]. Therefore, EVs play a key cell–cell communicator role not only in homeostasis but also in the cellular responses to various stimuli and diseases. Together with the available isolation of EVs from different body fluids such as plasma [24], bronchoalveolar lavage (BAL) [25], and sputum [26], these findings suggest that EVs have the potential for clinical application as future novel biomarkers and therapeutics in various diseases [17]. The aim of this review is to summarize the novel findings regarding the pathological roles of EVs in COPD development and discuss the potential clinical usefulness of EVs as biomarkers and therapeutic agents for the treatment of COPD.

## 2. Pathogenesis of COPD

COPD is a heterogeneous disease and its definition does not fully identify a variety of its features. In recent years, COPD have been classified into various distinct and overlapping phenotypes including clinical, physiological and radiologic manifestations, susceptibility to exacerbation and the asthma-COPD overlap syndrome, which suggest that there are complicated underlying mechanisms of the disease [27,28]. Among these mechanisms, the development of chronic inflammation in the lung in response to inhaled noxious particles and the accelerated aging of the lung are crucial hallmarks of COPD.

COPD is characterized by the chronic inflammation of the peripheral airways and lung parenchyma. The progression of COPD is associated with an increase of inflammation in the airway and alveolar wall [29]. Inhaled noxious substances initially activate pattern recognition receptors (PRRs), such as the Toll-like receptors expressed in alveolar macrophages, dendritic cells and epithelial cells, through the identification of microbial pathogen-associated molecular patterns [30,31]. In addition, these irritants induce the release of damage-associated molecular patterns such as uric acid and high-mobility group box 1 through the structural cell death, which also activates PRRs [32,33]. This results in the innate immune response, with increased numbers of neutrophils and macrophages. At the same time, the activation of structural cells such as epithelial cells and the increased secretion of mucus occurs in the lung. Subsequently, adaptive immune inflammation develops in the lung, with increased numbers of predominantly CD8^+^ T cells and CD4^+^ T helper 1 and 17 cells [30,34]. The resulting immune inflammation and activation of structural cells induce oxidative stress and many different inflammatory mediators such as cytokines, chemokines, proteases, inflammasomes and lipids. These mediators can interact with each other in a complex manner and are partly regulated by the activation of nuclear factor-κB [35]. These mediators also induce further inflammation in the lungs. Moreover, some proteases that degrade elastin fibers can contribute to the development of emphysema in lung [6].

COPD is also a disease of accelerated lung aging and cellular senescence [2,36]. COPD shows several aging-related hallmarks [37,38]: shorter telomere length [39]; cellular senescence in epithelial cells, endothelial cells and fibroblasts [40,41]; epigenetic alterations (e.g., such as those mediated by the sirtuin family of class III histone deacetylases) [42,43]; and mitochondrial dysfunction [44,45]. Excessive reactive oxygen species (ROS), which are formed during oxygen metabolism and induced by various stresses such as cigarette smoke, evoke the occurrence of molecular damage, resulting in cell cycle arrest and the accumulation of senescent cells [46]. Senescent cells affect their microenvironment by secreting various biochemical and inflammatory mediators which contribute to the senescence-associated secretory phenotype (SASP) [47]. In COPD pathogenesis, various cytokines (e.g., IL-1, IL-6, and IL-8), growth factors (e.g., insulin-like growth factor) and proteases (e.g., matrix metalloprotease) have been reported as SASP factors [48,49], which activate inflammatory and resident cells. Moreover, these activated cells in the airways of COPD patients also produce ROS. Therefore, the positive feedback loops of senescence, SASP, persistent inflammation, and ROS may be at least partly involved in COPD pathogenesis [49].

## 3. The Role of Epithelial EVs

Bronchial epithelial cells (BECs), which line the airway lumen, are among the primary sites of contact of environmental stimuli like cigarette smoke, and they perform a crucial role in maintaining normal airway homeostasis. The noxious effects of cigarette smoke induce airway epithelial injury and remodeling phenotypes such as squamous metaplasia, basal cell hyperplasia, alterations of cilia, and the suppression of the apical junctional barrier [50,51,52]. Injured lung epithelial cells release pro-inflammatory cytokines and chemokines as a source of various autocrine and paracrine factors, which influence the surrounding cells such as immune cells and subepithelial fibroblasts.

BECs are considered to be a major producer of EVs in the lung [53]. Epithelial cell-derived EVs are considered to be key players in the EV-mediated communication in the lungs. It has been reported that epithelial cell-derived exosomes have a size range of 30 to 100 nm and contain different membrane-tethered mucins of different sizes [54,55]. These surface mucins can provide sites of interaction between EVs and inhaled materials or host cells; hence, EVs may have important roles in homeostasis and innate defense. Moreover, the epithelial cell surface-associated mucin (MUC)-1, MUC-4, and MUC-16 contribute to the structuring of epithelial cell-derived EVs, which have a neutralizing effect on the human influenza virus [55]. MUC-1, MUC-4 and MUC-16, members of a family of large glycosylated proteins characterized by a variable number of tandem repeat motifs, can serve to keep epithelial surfaces moist and protect them from pathogens and environmental stimuli [56]. Therefore, these observations suggest that epithelial cell-derived EVs can be involved in the regulatory mechanisms of normal airway biology and perhaps in the pathogenesis of a wide range of lung diseases.

**Figure 1:**
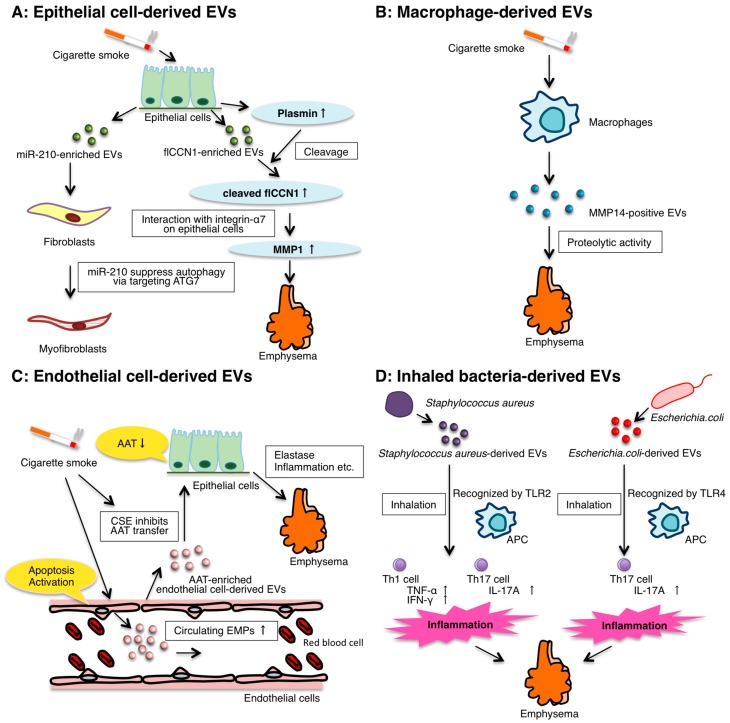
Summary of the reported functions of EVs in COPD. EVs are released from almost all cell types in the lung. EVs play a key cell-to-cell communicator role in the lung microenvironment. The following are the roles of the EVs released from different cell types in COPD pathogenesis. (**A**) Epithelial cell-derived EVs: CSE-induced human BEC-derived EV miR-210 promotes myofibroblast differentiation. Cleaved CCN1, a truncated form of full-length CCN1 in EVs by CSE, activates the secretion of MMP-1, which promotes the emphysematous changes; (**B**) Macrophage-derived EVs: CSE induces the release of macrophage-derived EVs with proteolytic activities attributed to MMP14; (**C**) Endothelial cell-derived EVs: the number of circulating EMPs increases in COPD patients and smokers. CSE inhibits AAT transport into epithelial cells via endothelial cell-derived EVs action; (**D**) Inhaled bacteria-derived EVs: *Staphylococcus aureus*-derived EVs induce Th1 and Th17 neutrophilic pulmonary inflammation. Repeated exposure to *Escherichia coli*-derived EVs induces neutrophilic inflammation, leading to emphysema. AAT, α1-antitrypsin; APC: antigen presenting cell; CCN1, CYR61/CTGF/NOV family 1; CSE, cigarette smoke extract; EMPs, endothelial microparticles; EVs, extracellular vesicles; microRNA, miR; MMP, metalloproteinase protein.

BEC-derived EVs can serve as effectors in the initiation and progression of COPD (Figure 1A). Moon et al. showed that exposure of epithelial cells to cigarette smoke extract (CSE) increases the protein concentration of the BEAS-2B human bronchial epithelial cell line-derived EVs and induces RAB27A, member RAS oncogene family, expression. Furthermore, RAB27A expression regulates lung epithelial cell-derived EV secretion in BEAS-2B cells and mouse lung tissues [57].

Our recent studies demonstrated that CSE-induced human BEC-derived EVs can cause airway fibrosis in COPD pathogenesis [58]. In this study, the evaluation of the modified EV and COPD lung samples indicated that cigarette smoke induced a relative upregulation of cellular and EV miR-210 expression in BECs. In co-culture assays, we showed that human BEC-derived EV miR-210 promotes the myofibroblast differentiation of lung fibroblasts (LFs). We found that miR-210 regulates autophagic processes by directly targeting ATG7, and the expression levels of miR-210 are inversely correlated with ATG7 expression in LFs. Importantly, autophagy induction was significantly decreased in LFs isolated from patients with COPD, and silencing ATG7 in LFs led to myofibroblast differentiation. In addition, we have reported the potential involvement of insufficient autophagy in COPD pathogenesis through the regulation of epithelial cell senescence [36]. Recently, we also reported that the insufficient mitophagy-mediated activation of the platelet-derived growth factor receptor (PDGFR)/phosphoinositide 3-kinase (PI3K)/AKT pathway, which is mainly attributed to reduced parkin RBR E3 ubiquitin protein ligase (PARK2) expression, is a potent underlying mechanism for myofibroblast differentiation [59]. These findings demonstrate that CSE triggers the modification of EV components and identifies BEC-derived miR-210 as a paracrine autophagy mediator of myofibroblast differentiation.

Moon et al. also reported one of the pathogenic factors of cigarette smoking-associated emphysema is CSE-induced lung epithelial cell-derived EVs [57]. They showed that CSE induced full-length CYR61/CTGF/NOV family1 (flCCN1)-enriched EVs. CCN1 is a cysteine-rich, extracellular matrix (ECM)-associated protein that plays crucial roles in numerous cellular activities including cell proliferation, adhesion, migration, differentiation, and apoptosis [1,60,61]. Although flCCN1 triggers inflammatory responses by mediating interleukin (IL)-8 release and the subsequent neutrophil recruitment, flCCN1-enriched EVs play crucial roles in lung homeostasis. This is because flCCN1 also facilitates the secretion of vascular endothelial growth factor (VEGF). Several studies have reported that the reduction of VEGF in the lung can induce the development of emphysema [62,63]. Interestingly, prolonged cigarette smoke exposure cleaved flCCN1 into its truncated form (cleaved CCN, cCCN1) in EVs by CSE-up-regulated plasmin that can cleave proteins between lysine-arginine and lysine-valine. cCCN1 interacts with integrin-α7 and activates the secretion of matrix metalloproteinase protein (MMP)-1 in lung epithelial cells. MMP-1 has been demonstrated to promote the emphysematous changes in the lung [64]. In addition, an elevated cCCN1 level was found in the BAL from mice with emphysematous changes after a 6-month exposure to CSE. Accordingly, the authors concluded that cCCN1 generated from flCCN1 by plasmin regulated cigarette smoke-induced emphysema.

## 4. The Role of Macrophage-Derived EVs

Macrophages play a pivotal role in the pathogenesis of COPD and have been recognized to be major effectors of the inflammatory response. The number of macrophages is remarkably increased in the airway, lung parenchyma, BAL fluid, and sputum of patients with COPD, and correlates with the severity of COPD [29,65,66]. Macrophages can secrete a range of pro- and anti-inflammatory mediators such as cytokines, proteases and protease inhibitors, as well as reactive oxygen species, which induce not only inflammation and emphysema but also wound repair, the control and resolution of inflammation, bacterial colonization, and corticosteroid resistance [67].

Macrophages are also major EV producers in the lung [53], which can be key players in maintaining homeostasis and immune cell production. For example, macrophage-derived EVs can induce the differentiation of naive monocyte recipient cells to macrophages through the transfer of miR-223, which is an important regulator of myeloid cell proliferation and differentiation [68]. In addition, macrophage-derived EVs contain major histocompatibility complex (MHC) class II and co-stimulatory molecules, suggesting a potential role in antigen presentation [11,69]. Moreover, the lung resident alveolar macrophages can secrete suppressor of cytokine signaling (SOCS)-1 and SOCS-3 in EVs [70].

To date, there are several reports on CSE-induced macrophage-derived EVs (Figure 1B). Li et al. reported that CSE increased the release of tissue factor-positive and procoagulant EVs [71]. Cordazzo et al. demonstrated that the CSE activates mononuclear cells via intracellular calcium mobilization and increases the release of macrophage-derived EVs with procoagulant and pro-inflammatory mediators, including IL-8, intercellular adhesion molecule-1, and monocyte chemoattractant protein-1 [72]. Furthermore, Li et al. have shown that CSE induced the release of macrophage-derived EVs with gelatinolytic and collagenolytic activities attributed to MMP14 [73]. MMP14 can be relevant to unstable atherosclerosis, and emphysematous lungs exhibit an upregulation of MMP14 in alveolar macrophages. These results indicate that MMP14-positive EVs may contribute to the instability of atherosclerotic plaques and emphysema in smokers. Although the roles of macrophage-derived EVs are not fully understood, it is likely that macrophages may contribute to COPD pathogenesis (e.g., inflammation and tissue injury) via releasing EVs.

## 5. The Role of Endothelial Cell-Derived EVs

Endothelial cells play a crucial role in the maintenance of vascular homeostasis [74]. Endothelial cells have many functions, including the reduction of vascular tone, the coordination of blood flow to control, and the modulation of hypoxic vasoconstriction [75,76]. The structural and functional impairment of small pulmonary arteries is commonly observed in the early stages of COPD, including vessel wall thickening, endothelial dysfunction, vascular smooth muscle proliferation, and inflammatory cell infiltration such as CD8^+^ T lymphocytes and macrophages in the vessel walls [77,78]. During disease progression, endothelial cells are considered be induced to undergo apoptosis [63,79]. Eventually, combinations of these pathological alterations may lead to pulmonary hypertension and right ventricular dysfunction. In addition, COPD patients have increased arterial stiffness, which can be an independent risk factor for cardiovascular events in association with atherosclerotic plaque formation [80,81,82].

Endothelial cells release different types of EVs including microparticles (also known as microvesicles), exosomes and apoptotic bodies. Endothelial microparticles (EMPs) are shed into the circulation from activated or apoptotic endothelial cells and carry endothelial proteins such as platelet endothelial cell adhesion molecule-1 (CD31), vascular endothelial cadherin (CD144) and E-selectin (CD62E). EMPs play an important role in coagulation, inflammation, endothelial function, and angiogenesis. Moreover, circulating EMPs increase in response to various stresses such as cigarette smoke, endotoxin, and cyclic stretching [83], and in a wide range of diseases such as coronary artery disease, diabetes, atherosclerosis, hypertension, and renal failure [84]. Therefore, EMPs play an important role in disease pathogenesis, and can be biomarkers of endothelial cell apoptosis or activation.

Other groups have reported an increase in the numbers of circulating EMPs in response to cigarette smoke exposure and COPD in patients (Figure 1C). Heiss et al. showed that the brief exposure of nonsmokers to secondhand smoke increased the number of circulating EMPs resulting from acute injury that persisted for at least 24 h [83]. Gorden et al. described an increase in the number of circulating EMPs in smokers with early emphysema [85,86]. Moreover, some types of EMPs are also increased in the circulation of patients with COPD and may play a role in linking COPD to cardiovascular comorbidities [85,87]. Furthermore, the increase in EMPs is related to the Global Initiative for Chronic Obstructive Lung Disease (GOLD)-disease stage progression in COPD as well as the degree of emphysema. In addition, the increased endothelial EVs may reflect pulmonary capillary endothelial injury because EVs possess the angiotensin-converting enzyme. Recently, in a paper by Strulovic-Barel et al., the same research group presented that the levels of total and apoptotic EMPs remain elevated over 12 months significantly in COPD smokers who quit smoking although in healthy smokers who quit smoking, the levels of total and apoptotic EMPs return to the levels of non-smokers [88].

Endothelial cell-derived EVs including EMPs are involved in intracellular communication as a paracrine or endocrine factor and can contribute to the pathogenesis of COPD. Lockett et al. suggested that lung endothelial cells transport α1-antitrypsin (AAT) into the alveolar epithelial cells via endothelial cell-derived EVs [87] (Figure 1C). In general, AAT is a protease inhibitor secreted by hepatocytes into the systemic circulation that inhibits the action of the proteolytic enzyme elastase and the proteases trypsin, chymotrypsin, and thrombin that are produced. AAT is internalized by lung epithelial cells to protect the lung from elastase, inflammation, and endothelial cell apoptosis. Although AAT deficiency is generally considered to be rare, approximately 1% to 5% of patients with COPD are estimated to have an AAT deficiency [89]. In addition, Lockett et al. showed that CSE decreased the levels of secreted AAT from endothelial cells. These results suggest that CSE inhibits AAT transport into epithelial cells via endothelial cell-derived EVs activity, which has a potential role in COPD pathogenesis.

## 6. The Role of Inhaled Bacteria-Derived EVs

Similar to mammalian cells, all gram-negative and some gram-positive bacteria (e.g., *Staphylococcus aureus*, *Bacillus species*, and *Streptococcus pneumonia*) produce bacteria-derived EVs [90,91]. Gram-negative bacteria-derived EVs are produced from the outer membrane of the cell envelope and are thus usually called outer membrane vesicles. In contrast, gram-positive bacteria have a thick peptidoglycan cell wall without their outer membrane, which may have prevented the existence of their EVs until recently. The contents of gram-negative and gram-positive bacteria-derived EVs have a wide variety of molecules, such as proteins, lipids, DNAs, RNAs and various virulence factors, which can play important physiological and pathological roles in bacteria-bacteria and bacteria-host interactions via EVs [90,92,93]. In addition, they have been found in various circumstances such as in biofilm, water drains, soil, house dust and blood from patients with severe infectious diseases [94,95,96].

Recently, the research group of Yong Song Gho reported the relationship between bacteria-derived EVs in indoor dust and neutrophilic pulmonary inflammation (Figure 1D). In 2010, they showed that the intraperitoneal injection of *Escherichia coli*-derived EVs induced systemic inflammation that mimicked sepsis after entering the bloodstream [97]. Then in 2012, the same group showed that the repeated inhalation of *Staphylococcus aureus*-derived EVs induced a pulmonary inflammatory response of neutrophil infiltration. The repeated inhalation of the EVs also induced both interferon (IFN)-γ and IL-17 cytokines in the lung. In addition, the immune responses induced by the EVs were dependent on Toll-like receptor 2 (TLR2) signaling. Therefore, *S. aureus*-derived EVs could induce T helper, type 1 (Th1) and Th17 neutrophilic pulmonary inflammation, mainly in a TLR2-dependent manner. Furthermore, in 2013, airway exposure to EVs from indoor dust, which contains bacteria (mainly *E. coli*)-derived-EVs, also induced neutrophilic inflammation and subsequent emphysema in the lungs [96]. Thus, these data indicate that *E. coli*-derived EVs, which are present in indoor dust, can be one of the factors involved in the pathogenesis of neutrophilic inflammation-induced obstructive airway diseases such as COPD and asthma. In 2015, the research group showed that repeated exposure to *E. coli*-derived EVs induces neutrophilic inflammation, thereby leading to emphysema mainly in an IL-17a-dependent manner [98]. Moreover, the *E. coli*-derived EV uptake by inflammatory cells such as macrophages is dependent on both lipopolysaccharide and TLR4 signaling. Therefore, EVs from gram-negative bacteria can cause COPD.

## 7. EVs in COPD Exacerbation

The acute exacerbation of COPD is an event characterized by the worsening of the patient’s respiratory symptoms beyond the normal day-to-day variations, which leads to a change in their medication [1]. Not only does COPD exacerbation have a devastating impact on a patient’s quality of life, but it also causes morbidity and mortality. The major causes of COPD exacerbation are respiratory viral and bacterial infections [99]. Exacerbations can also occur from inhaling environmental pollutants or an unknown etiology. Although there are few reports of EV-induced COPD exacerbation, EVs may influence this process.

It has been reported that endothelial injury occurs during exacerbation, and to clarify the influence of COPD exacerbation on the endothelium, Takahashi et al. investigated EMPs [85]. In their report, cluster of differentiation (CD)-144^+^, CD-31^+^, and CD-62E^+^ EMPs were significantly higher in patients during COPD exacerbation than those in stable patients, reflecting endothelial damages during the COPD exacerbation. It has been speculated that increased specific EMPs could be a possible predictive biomarker for COPD exacerbation. It has also been reported that adenosine triphosphate (ATP) levels are increased in the airways of patients with COPD and asthma. Eltom et al. have reported that respiratory infections can trigger the release of EVs in mice and humans [100]. Then, following ATP-mediated activation via purinergic receptor P2X7, the EVs release IL-1b and IL-18 in a P2X7/caspase-1 axis-dependent manner, thereby exacerbating neutrophilia. Therefore, these authors considered EVs and their associated signaling pathways as possible mechanisms underlying the exacerbation of respiratory diseases including COPD by infections.

## 8. Circulating EVs and miRNAs as Potential Biomarkers for COPD

According to the GOLD strategy for the diagnosis and management of COPD, the standard diagnostic criterion for COPD should be based on the persistent airflow limitation, which is defined by the presence of a post-bronchodilator ratio of forced expiratory volume in 1 s to forced vital capacity (FEV1/FVC) < 0.70 (FEV1, forced expiratory volume in 1 s; FVC, forced vital capacity) [1]. The severity of the airflow limitation should be evaluated by the predicted percentage FEV1 using spirometry. Although spirometry is a simple test, the presence of COPD may be underdiagnosed in younger patients but overdiagnosed in older patients [101]. In addition, COPD is now widely recognized as a complex heterogeneous disease. In the assessment of patients with COPD, it is important to identify clinical phenotypes based on the prognosis and response to therapy for selecting the optimal treatment. Therefore, additional biomarkers for COPD are needed to complement the information obtained from spirometry.

Recently, EVs have been identified as a new disease biomarker for the following reasons. First, EVs reflect the physiological state and microenvironment of their cells of origin, and most cells secrete EVs containing specific proteins and nucleic acids [22,23,102]; Second, EVs are found in the blood and other body fluids; Third, EVs are very stable in the extracellular environment after their release from cells because of the phospholipid bilayer. In fact, numerous EV proteins and miRNAs have already been identified as potentially useful biomarkers for various diseases, especially in cancer detection [21,103,104,105].

Circulating EMPs are now being analyzed to evaluate the endothelial damage in patients with COPD and their clinical correlations (Table 1). Several studies have reported that some types of EMPs represent potential new biomarkers for COPD. Thomashow et al. reported that CD31^+^ EMPs, reflecting endothelial cell apoptosis, were elevated in mild COPD and emphysema. In contrast, CD62E^+^ EMPs, indicating endothelial activation, were elevated in severe COPD and hyperinflation [106]. Takahashi et al. showed that CD144^+^ (the most specific marker for endothelial cells), CD31^+^, and CD62E^+^ EMPs were significantly higher in patients with stable COPD than in the healthy non-COPD volunteers [85]. In addition, Lacedonia et al. reported a negative correlation between the number of EMPs in the sputum and the FEV1 [107].

Circulating miRNAs are also stable and protected from ribonucleases through an association with lipoproteins such as high-density lipoprotein, RNA-binding proteins such as argonaute-2, and nucleophosmin 1; alternately, they can be transported inside EVs [108,109,110]. Previous studies have reported that various miRNAs are involved in the development and progression of COPD [111]. Studies also analyzed the miRNA profile in the plasma or sputum for risk prediction and diagnosis of COPD (Table 1). Xie et al. suggested that the levels of serum miR-21 and miR-181a and their ratio have potential biomarker utility for predicting the development of COPD in heavy asymptomatic smokers [112]. In this study, 41 healthy controls, 40 asymptomatic heavy smokers and 49 COPD patients were analyzed for serum miRNA profiles. The results showed that the levels of serum miR-21 and miR-181a in asymptomatic heavy smokers and COPD patients were significantly higher than in healthy control patients. They also showed that the area under the ROC curve (AUC) was 0.815 with a sensitivity of 73.2% and specificity of 75.0% for miR-21, the AUC was 0.767 with a sensitivity of 62.5% and specificity of 75.6% for miR-181a, and the AUC was 0.910 with a sensitivity of 85.4% and specificity of 85.0% for the ratio of serum miR-21 to miR-181a in COPD patients. Akbas et al. reported that the levels of serum miR-20a, miR-28-3p, miR-34c-5p and miR-100 were up-regulated, whereas miR-7 was down-regulated compared with healthy controls [113]. Pottelberge et al. investigated miRNA expression in the induced sputum of COPD patients [114]. They performed an initial screening cohort consisting of 10 never-smokers, 10 current smokers and 12 current smokers with COPD and a validation cohort consisting of 10 never-smokers, 10 current smokers, 10 current smokers with COPD and 11 ex-smokers with COPD. The results showed that let-7c and miR125b were significantly down-regulated in the sputum of COPD patients, compared with healthy controls. Finally, Pinkerton et al. reported that let-7a, miR-328, and miR-21 have potential biomarker utility for COPD in exhaled breath condensates [115]. Taken together, these data demonstrated that EVs and circulating miRNAs have the potential to be attractive biomarkers of COPD, although further investigations are needed, and several technical and scientific obstacles in the way of clinical application must be overcome.

## 9. EVs as Potential Therapeutics for COPD

The contributions of EVs to COPD pathogenesis highlight their potential as novel therapeutic targets. In general, there are two therapeutic strategies targeting EVs: (1) Eliminating the EVs that contain nucleic acids or proteins as mediators of intracellular communication involved in disease pathogenesis; and (2) Using EVs as a source of lung regenerating or immune modulating agents. Eliminating EVs could be achieved by several different therapeutic approaches, including capturing the circulating EVs, disrupting EVs uptake by recipient cells, and inhibiting EVs production or secretion [103,116]. However, considering the latter strategy, the best examples are treatment with mesenchymal stem/stromal cells (MSCs) derived-EVs [17]. MSCs are multipotent and non-hematopoietic cells with the potential of being able to differentiate into several cell types. Recently, it has been discovered that MSCs have the potential ability to orchestrate tissue regeneration, anti-inflammation and/or immunosuppression. Thus far, numerous studies have suggested that MSC-derived EVs seem to have the same functions. Although there are no studies using the above two strategies regarding COPD treatment, the clinical effects of some EVs have already been studied in ongoing phase I and II trials for cancer and transplantation treatments [117,118,119,120]. For example, the therapy of MSC-EVs to patients with steroid-refractory graft-versus-host disease (GvHD) was conducted [120]. During treatment, no significant side effects were observed. Interestingly, the GvHD-symptoms such as diarrhea and skin ulcers were significantly improved, and the patient was stable for more than 4 months after MSC-EVs treatment [120].

Nonetheless, the following problems need to be clearly addressed before the clinical use of EVs. First, it is difficult to identify the EVs secreted from specific cells in the numerous numbers of EVs in body fluids using the currently available technologies. Second, the definition and standard isolation methods for EVs are not fully established. Third, the exact mechanism of the interaction between EVs and recipient cells and EVs distribution in the body is not fully understood. Therefore, further investigation is crucially needed to establish the disease-specific effects of EV treatment.

## 10. Conclusions

EVs have been recognized as an emerging novel cell–cell communication tool in numerous physiological and pathological processes. Investigating the role of EVs is an emerging and rapidly progressing area of research, particularly regarding the lungs. The findings described in this article confirm that EVs play a pivotal role in the pathogenesis of COPD, and additional research on the pathogenesis of this disease could contribute to the development of novel therapies targeting EVs. Furthermore, the stability of EVs in the extracellular fluids and the disease-specific molecules that EVs contain suggest they could have great advantages as disease biomarkers. Further studies of the precise pathological functions and roles of EVs are necessary for their clinical application as biomarkers and therapeutic agents.

## Figures and Tables

**Table 1:** Circulating microRNAs and EVs as potential biomarkers for COPD.

Body Fluids	Potential Biomarkers	Detection Methods	References
miRNAs			
serum	miR-21/miR-181a ratio	realtime PCR	[112]
serum	upregulated: miR-7 downregulated: miR-20a, miR-28-3p, miR-34c-5p, miR-100	realtime PCR	[113]
sputum	downregulated: let-7c, miR-125b	realtime PCR	[114]
exhaled breath condensates	dounregulated: let-7a, miR-21, miR-328	realtime PCR	[115]
EVs			
plasma	CD31^+^ EMPs, CD62E^+^/CD31^+^ EMPs ratio	flow cytometry	[86,88]
plasma	CD31^+^ EMPs, CD62E^+^ EMPs	flow cytometry	[106]
plasma	CD144^+^ EMPs, CD31^+^ EMPs, CD62E^+^ EMPs	flow cytometry	[85]
sputum	CD31^+^ EMPs, CD66^+^ EMPs, CD235ab^+^ EMPs	flow cytometry	[107]
